# Ferroptosis and musculoskeletal diseases: “Iron Maiden” cell death may be a promising therapeutic target

**DOI:** 10.3389/fimmu.2022.972753

**Published:** 2022-10-11

**Authors:** Yili Zhang, Xinyi Huang, Baoyu Qi, Chuanrui Sun, Kai Sun, Ning Liu, Liguo Zhu, Xu Wei

**Affiliations:** ^1^ School of Traditional Chinese Medicine and School of Integrated Chinese and Western Medicine, Nanjing University of Chinese Medicine, Nanjing, China; ^2^ School of Traditional Chinese Medicine, Beijing University of Chinese Medicine, Beijing, China; ^3^ Wangjing Hospital, China Academy of Chinese Medical Sciences, Beijing, China

**Keywords:** regulated cell death, immunology, oxidative stress, inflammation, mechanism

## Abstract

Ferroptosis is a novel form of cell death precisely regulated by iron metabolism, antioxidant processes, and lipid metabolism that plays an irreplaceable role in the development of many diseases. Musculoskeletal disorders (MSKs), including osteoporosis, osteoarthritis, rheumatoid arthritis, intervertebral disc degeneration, sarcopenia, and rhabdomyolysis, have become one of the most common causes of disability and a major burden on public health and social care systems. The mechanism of ferroptosis in MSKs has recently been elucidated. In this review, we briefly introduce the ferroptosis mechanism and illustrate the pathological roles of ferroptosis in MSKs with a focus on how ferroptosis can be exploited as a promising treatment strategy. Notably, because the toxicity of compounds that inhibit or induce ferroptosis in other organs is largely unknown, ferroptosis appears to be a double-edged sword. We point out that more research is needed in the future to verify the therapeutic effects based on ferroptosis in MSKs.

## Introduction

Classically, the regulation of cell death is assumed to be achieved by two main models: accidental cell death (ACD) and regulated cell death (RCD) (1). ACD is usually triggered by an unexpected injury or attack, which overwhelms any possible molecular control mechanism (2). Whereas, the process of RCD, manifested as classical apoptosis, is regulated by a number of molecules with genetically defined effector and precise signaling cascades involving unique immunological, functional, and biochemical consequences. A growing body of evidence in recent years reveals that many nonapoptotic forms of RCD, including pyroptosis, necrosis, autophagy, and ferroptosis, contribute to various pathologies in humans (3).

**Figure 1:**
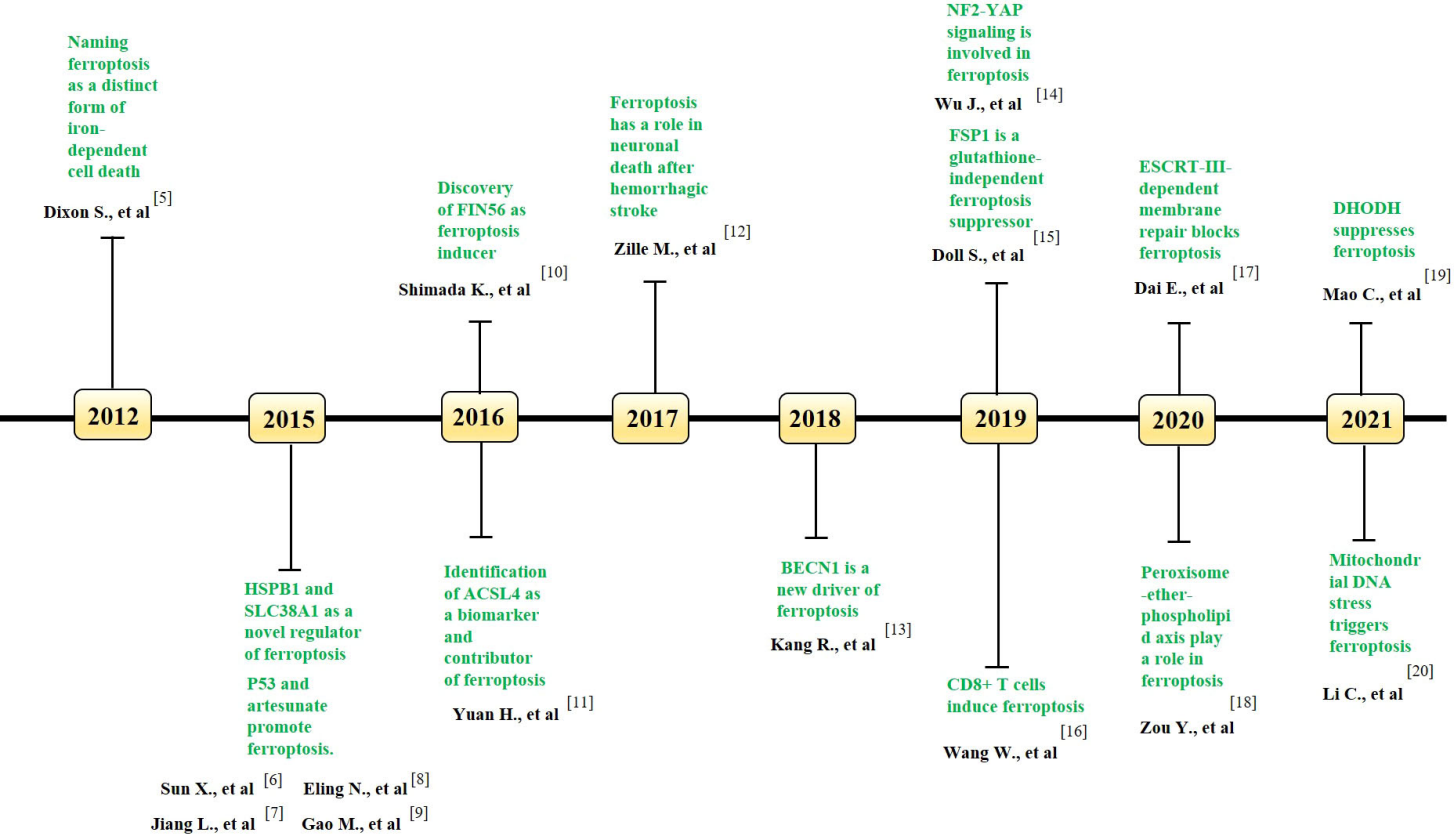
Key milestones in the literature of ferroptosis over time. The key discoveries related to ferroptosis in each year is indicated. GPX4, Glutathione peroxidase 4; HSPB1, heat shock protein beta-1; SLC38A1, solute carrier family 38 member 1; FIN56, Ferroptosis inducing 56; ACSL4, acyl-coenzyme A synthetase long-chain family member 4; BECN1, beclin 1; NF2, neurofibromin 2; YAP, Yes-associated protein; FSP1, ferroptosis suppressor protein 1; ESCRT, endosomal sorting complexes required for transport; DHODH, dihydroorotate dehydrogenase.

**Figure 2:**
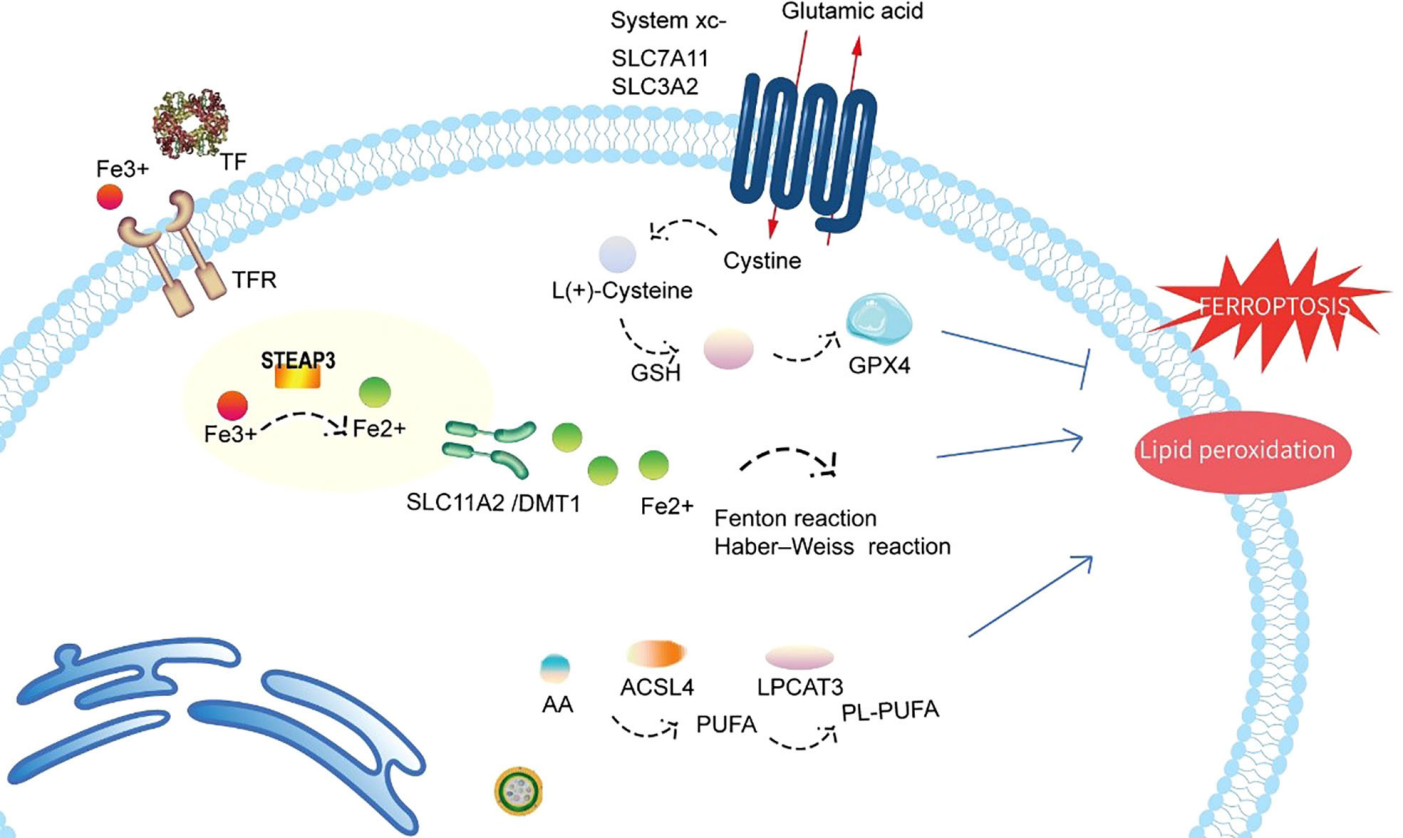
Schematic representation of the mechanism of ferroptosis. Ferroptosis is a novel form of cell death precisely regulated by iron metabolism, antioxidant processes, and lipid metabolism. Fe3+ imported through the transferrin receptor is converted to Fe2+ in endosomes and released from endosomes by DMT1 (also known as SLC11A2). Excess Fe2+ can induce ferroptosis through reactive oxygen species generated by the Fenton reaction and Haber–Weiss reaction. GPX4 is the major endogenous mechanism that suppresses lipid peroxidation. Cystine enters cells through system Xc-, and cystine is subsequently converted to cysteine, which generates glutathione (GSH), a cofactor for GPX4. Both LPCAT3 and ACSL4 also exert direct or indirect actions on lipid peroxidation of membrane PUFAs. TF, transferrin; TFR, transferrin receptor; STEAP3, six-transmembrane epithelial antigen of prostate 3; SLC11A2/DMT1, divalent metal transporter 1; SLC7A11, solute carrier family 7 member 11; SLC3A2, solute carrier family 3 member 2; system xc-, cystine/glutamate antiporter system; GPX4, glutathione peroxidase 4; GSH, glutathione; PL-PUFA, phospholipid-bound polyunsaturated fatty acids; PUFA, polyunsaturated fatty acid; ACSL4, acyl-coenzyme A synthetase long-chain family member 4; LPCAT3, lysophosphatidylcholine acyltransferase 3.

**Figure 3:**
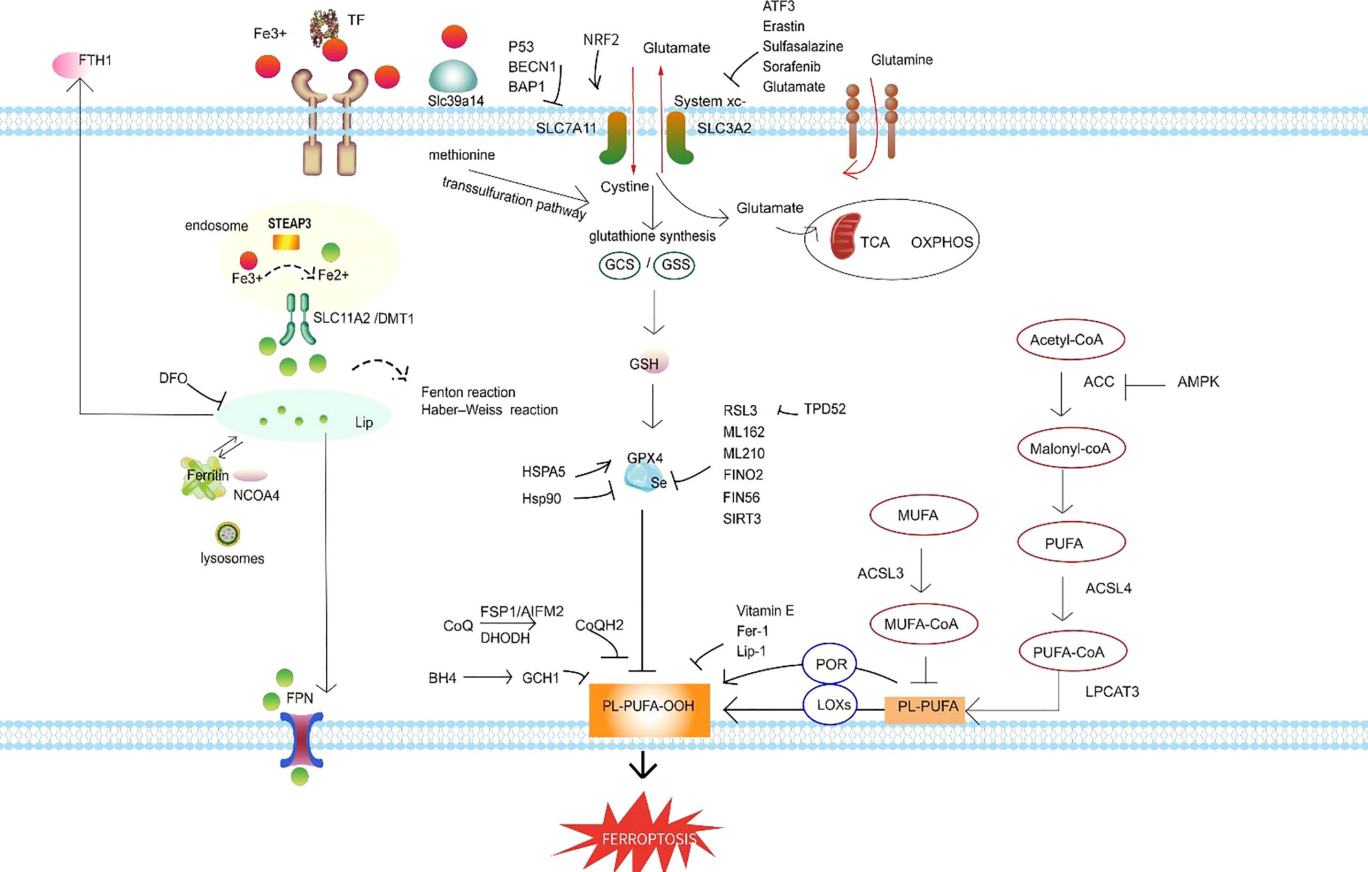
Ferroptosis regulatory pathways. The regulatory pathways of ferroptosis can be roughly classified into three types. The first involves iron metabolism, including the regulation of ferritin degradation by NCOA4. The second is the GSH/GPX4 pathway, which includes system xc inhibition, the transsulfuration pathway, and the glutamine pathway. The third is lipid metabolism, including ACSL4, ACSL3, and LPCAT3, which are related to lipid regulation and ferroptosis. In addition, FSP1 inhibits ferroptosis independently of GSH, and the BH4–GCH1 axis effectively inhibits lipid peroxidation and thereby defends against ferroptosis. Abbreviations: TF, transferrin; FTH1, ferritin heavy chain 1; DFO, defetoxamine; LIP, labile iron pool; NCOA4, nuclear receptor coactivator 4; FPN, ferroportin; SLC11A2/DMT1, divalent metal transporter 1; STEAP3, six-transmembrane epithelial antigen of prostate 3; NRF2, NF-E2–related factor 2; SLC7A11, solute carrier family 7 member 11; SLC3A2, solute carrier family 3 member 2; system xc-, the cystine/glutamate antiporter system; Slc39a14, the zinc transporter Zip14; BECN1, beclin 1; BAP1, BRCA-1–associated protein; ATF3, recombinant activating transcription factor 3; GCS, glutamylcysteine synthetase; GSS, recombinant glutathione synthetase; TCA, tricarboxylic acid; OXPHOS, oxidative phosphorylation; GPX4, glutathione peroxidase 4; GSH, glutathione; HSPA5, heat shock protein A5; HSP90, heat shock protein 90; RSL3, RAS-selective-lethal-3; FINO_2_, an endoperoxide-containing 1,2-dioxolane; FIN56, ferroptosis-inducing 56; SIRT3, sirtuin 3; TPD52, tumor protein D52; Fer-1, ferrostatin-1; Lip-1, liproxstatin-1; FSP1, ferroptosis suppressor protein 1; AIFM2, apoptosis-inducing factor mitochondria-associated 2; BH4, tetrahydrobiopterin; GCH1, GTP cyclohydrolase-1; PL-PUFA-OOH, lipid peroxides; POR, NADPH-cytochrome P450 reductase; LOXs, lipoxygenases; PUFA, polyunsaturated fatty acid; MUFA, monounsaturated fatty acid; PL-PUFA, phospholipid-bound polyunsaturated fatty acids; ACSL4, acyl-coenzyme A synthetase long-chain family member 4; LPCAT3, lysophosphatidylcholine acyltransferase 3; ACC, acetyl CoA carboxylase; AMPK, AMP-activated protein kinase.

In 2002, Dolma et al (4). performed a study of the Epithelial cells expressing oncogenic Ras (RasV12) cell line and found that erastin, a novel compound, initiates a cell death process displaying no apoptotic features, such as fragmented nuclei, DNA laddering, and activated caspase 3, which later came to be known as ferroptosis. The cell death model ferroptosis was officially recognized as a novel form of RCD in 2012 (5) (see **Figure 1** for a glossary of key terms in ferroptosis). Ferroptosis involves many pathophysiological processes characterized by lipid peroxidation caused by the accumulation of iron-dependent reactive oxygen species (ROS) in cells. The mechanisms and regulatory pathways of ferroptosis are complicated and involve a variety of signaling molecules and metabolic pathways (**Figures 2**, **3**). Of note, ferroptosis participates in the occurrence and development of various diseases.

Aging, a natural and complex physiological process, is generally considered the greatest risk factor for many neurodegenerative, metabolic, cardiovascular, and musculoskeletal disorders (MSKs). Among these conditions, MSKs, including a wide range of inflammatory and degenerative diseases such as osteoporosis, osteoarthritis (OA), rheumatoid arthritis (RA), and sports injuries, are some of the most common causes of chronic disability worldwide (21). With a large number of cases, MSKs remain a disease of international concern, which has resulted in an enormous global disease burden (22). Therefore, therapeutic targets linking aging and disease may extend the healthy life span of patients and limit healthcare costs.

In recent years, neurological, cardiovascular, and neoplastic diseases have been the focus of both ferroptosis research and clinical applications (23–25). As a hot topic, ferroptosis is now known to play a critical role in multiple systems or organs (26). Interestingly, a growing body of evidence has recently uncovered links between ferroptosis and MSKs. However, there are relatively few reviews in the field of MSKs, which gives us an opportunity to remedy this major deficiency. Herein, we summarize the basic pathological features of ferroptosis and discuss its potential role in the pathophysiology of these diseases and associated complications.

## Mechanisms and regulation of ferroptosis

The mechanism of ferroptosis has been summarized almost perfectly (27, 28). In general, ferroptosis has its own morphological, biochemical, and genetic characteristics. Morphologically, mitochondrial shrinkage, which involves decreased mitochondrial cristae and increased membrane density, is a characteristic feature of ferroptosis (29). Biochemically, ferroptosis is activated by the formation of iron-dependent ROS, which can be inhibited by antioxidants and iron chelators rather than apoptosis, necrosis, or autophagy inhibitors (5). The broad biological processes include iron metabolism, antioxidant processes, and lipid metabolism.

## Iron metabolism

All these observations allowed the identification of iron as a critical cofactor in various biochemical enzyme-catalyzed reactions involved in the physiological regulation of oxygen transport, energy metabolism, DNA synthesis, and repair. Iron has implications in several disorders of phosphate and bone metabolism (30). Hydroxyl radicals (HO·), generated by Fe2+ through the Fenton reaction, might be associated with damage to proteins, lipids, and DNA.

The maintenance of iron homeostasis is crucial for the normal function of cells. Several studies have found that abnormal iron metabolism as a result of iron overload is the main characteristic of ferroptosis. Circulating iron binds to transferrin receptor 1 (TFR1) on the cell membrane, and in this reaction, ferric iron is reduced to ferrous iron by the six-transmembrane epithelial antigen of prostate 3 (STEAP3). Subsequently, divalent iron is released by divalent metal transporter 1 (DMT1) into the labile iron pool (LIP) in the cytoplasm. Of note, lysosomes, which store large quantities of LIP, are considered the main organelles responsible for cellular ferroptosis and represent promising potential disease targets (31). Nuclear receptor coactivator 4 (NCOA4)–mediated ferritinophagy increases the degradation of ferritin by lysosomes, reduces iron storage, and promotes ferroptosis (32). Excess bivalent iron is then transported extracellularly by ferroportin 1 (FPN1) and stored in ferritin heavy chain 1 (FTH1) and ferritin light chain 1 (FTL1). In addition, both treatments with the ferroptosis inhibitor ferrostatin-1 (Fer-1) and hepatocyte-specific knockout of the metal transporter Slc39a14 significantly reduce iron overload-induced liver ferroptosis in transferrin knockout mice (Trf-LKO) mice (33).

From a physiopathological point of view, ferritin is a strong buffer involved in regulating iron deficiency and maintaining homeostasis (34). The regulation of mitochondrial iron metabolism is assumed to be achieved by mitochondrial ferritin, and its overexpression can reverse ferroptosis induced by erastin (35). Under pathological conditions, during the process of Fenton and Haber–Weiss reactions, iron overload induces ferroptosis by producing high concentrations of ROS (36, 37).

Deferoxamine (DFO), an iron chelator, works by inhibiting ferroptosis as a result of intracellular iron overload (38). In addition, mitochondrial transferrin mitoferrin 1/2 is destroyed on the inner mitochondrial membrane, which consequently results in abnormal iron metabolism in the mitochondria (39). In summary, increased iron intake, reduced stable iron, and decreased iron outflow ultimately stimulate oxidative damage and lead to ferroptosis.

## Antioxidant systems

### Glutathione peroxidase 4

Glutathione peroxidase 4 (GPX4), one of the eight glutathione (GSH) peroxidases, serves as the primary intracellular antioxidant buffer, which plays an indispensable role in antioxidant effects and ferroptosis regulation. It has been demonstrated that selenium (Se) can improve GPX4 expression (40). The conversion of reduced GSH to oxidized glutathione (GSSG) is achieved by either the conversion of free hydrogen peroxide to water or the reduction of lipid peroxides (L-OOH) to lipid hydroxyl derivatives (LOH), and both are essential for the maintenance of cellular redox homeostasis (41). In addition, both the GSH/GSSG ratio and GSH reflect the oxidation resistance and are therefore associated with ferroptosis (42).

The overexpression of mitochondrial GPX4 inhibits mitochondrial oxidative stress and mitochondrial-dependent apoptosis, whereas its deficiency leads to massive cell death (43). As a substrate of GPX4, RAS-selective-lethal-3 (RSL3) serves as a ferroptosis-induced molecule that works by binding to GPX4 in an iron-, mitogen-activated protein kinase kinase (MEK)-, and ROS-dependent manner. Genetically enhancing tumor protein D52 (TPD52)–dependent lipid storage prevents RSL3-induced lipid peroxidation and subsequent ferroptosis *in vitro* and *in vivo* (44). However, the overexpression of GPX4 may induce resistance to RSL3. GPX4 activity is also inhibited directly or indirectly by other chemical compounds, such as ML162, ML210, Diphenyleneiodonium chloride (DPI) compounds, buthionine sulfoximine, sirtuin 3, FINO_2_, and FIN56 (45, 46). In addition, studies have shown that the activation of transcription factor 4 (ATF4) leads to the induction of HSPA5, which, in turn, binds to GPX4 and prevents GPX4 protein degradation and subsequent lipid peroxidation (47). On the basis of the existing scientific research, HSP90 family members may act on GPX4, which results in inhibition of the antioxidant capacity of GPX4 by inhibiting its activity (48).

### Cystine/glutamate antiporter system

The selective inhibition of the cystine/glutamate antiporter system (system xc-) works by decreasing intracellular GSH, exacerbating the accumulation of ROS, and eventually leading to ferroptosis (49). System xc- is composed of solute carrier family 3 member 2 (SLC3A2) and solute carrier family 7 member 11 (SLC7A11), and the major negative effect of system xc- on ferroptosis regulation appears to be due to its crucial role in the synthesis of the antioxidant GSH, which allows the exchange of exogenous cystine with glutamate in a 1:1 ratio. GSH is then synthesized by cysteine, which is degraded from cystine (50, 51). In addition, studies have verified that many exogenous compounds or endogenous genes can activate or inhibit system xc-. Genetically, system xc- could be positively regulated by NF-E2–related factor 2 (NRF2) and ubiquitin thioesterase. In addition, BRCA-1–associated protein (BAP1) and p53 can negatively regulate system xc- (52). The ATF3 enhances the ferroptosis induced by erastin *via* the repression of system xc- (53), whereas AMP-activated protein kinase (AMPK)–mediated beclin 1 (BECN1) phosphorylation increases ferroptosis by directly inhibiting system xc- activity (54). Radiotherapy and immunotherapy enhance lipid oxidation and the ferroptosis of tumor cells by synergistically suppressing SLC7A11 (55). Sorafenib and sulfasalazine inhibit system xc- function and induce ferroptosis (56, 57), whereas GDF15 knockdown facilitates ferroptosis induced by erastin *via* the attenuation of SLC7A11 expression (58). Moreover, P53 can enhance ferroptosis by inhibiting the expression of SLC7A11 (59).

Cysteine availability restricts GSH biosynthesis, whereas cysteine starvation induces GSH depletion and ferroptosis. When the available cysteine is limited, some cells utilize the transsulfuration pathway to transform methionine to cysteine (60). Glutamate is also an important regulator of ferroptosis. At high concentrations, this compound suppresses system xc- and triggers ferroptosis. Glutamine degradation (*via* glutaminolysis) fuels the tricarboxylic acid (TCA) cycle and provides the basis for necessary biosynthetic processes, such as lipid biosynthesis (61).

### Lipid metabolism

Lipid peroxidation is considered the primary factor in ferroptosis. Lipid peroxides have the ability to destroy the stability of the lipid bilayer and thus the disintegration of cell membranes (62). Researchers have suggested that polyunsaturated fatty acids (PUFAs) are susceptible to lipid peroxidation, possibly due to the presence of highly active hydrogen atoms in methylene bridges. Hydroxyl radicals exert direct effects on the formation of lipid peroxides by interacting with PUFAs in membrane phospholipids, which then attack the cytomembrane and trigger ferroptosis (63). Furthermore, nucleic acids and proteins react with derivatives produced by the decomposition of lipid peroxides, such as 4-hydroxynonenal (4-HNE) and malondialdehyde (MDA), which could also lead to cell destruction (64). These derivatives, which act as markers, could also be useful for the detection of ferroptosis and lipid peroxidation. NADPH (nicotinamide adenine dinucleotide phosphate)–cytochrome P450 reductase transfers electrons from NAD(P)H to oxygen to generate hydrogen peroxide, which subsequently reacts with iron to generate reactive hydroxyl radicals for the peroxidation of the PUFA chains of membrane phospholipids, thereby disrupting membrane integrity during ferroptosis (65). In addition, ferroptosis is promoted by LOX-catalyzed lipid hydroperoxide generation in cellular membranes (66). Mechanistically, AMPK regulates ferroptosis through acetyl-CoA carboxylase (ACC) and PUFA biosynthesis (67). Exogenous monounsaturated fatty acids (MUFAs) potently inhibit ferroptosis. This effect requires MUFA activation by acyl-coenzyme A synthetase long-chain family member 3 (ACSL3) and is independent of lipid droplet formation (68). In addition, lipid peroxidation is inhibited by Fer-1, liproxstatin-1 (Lip-1), and vitamin E, which are free radical scavengers that reduce lipid peroxidation and effectively block ferroptosis (69, 70). Ferroptosis suppressor protein 1 (FSP1) suppresses ferroptosis independent of GSH. In the presence of NADPH, FSP1 reduces ubiquinone, also called coenzyme Q10 (CoQ10), to ubiquinol, which can reduce lipid peroxidation and promote ferroptosis (71). The synthesis and recycling of tetrahydrobiopterin (BH4) is a dynamic process, and GTP cyclohydrolase-1 (GCH1) is the rate-limiting enzyme in the biosynthesis of BH4. GCH1-mediated BH4 production prevents ferroptosis by inhibiting lipid peroxidation, which indicates that BH4 exhibits antioxidant activity during cell death (72).

## Ferroptosis and MSKs

### Osteoporosis

**Figure 4:**
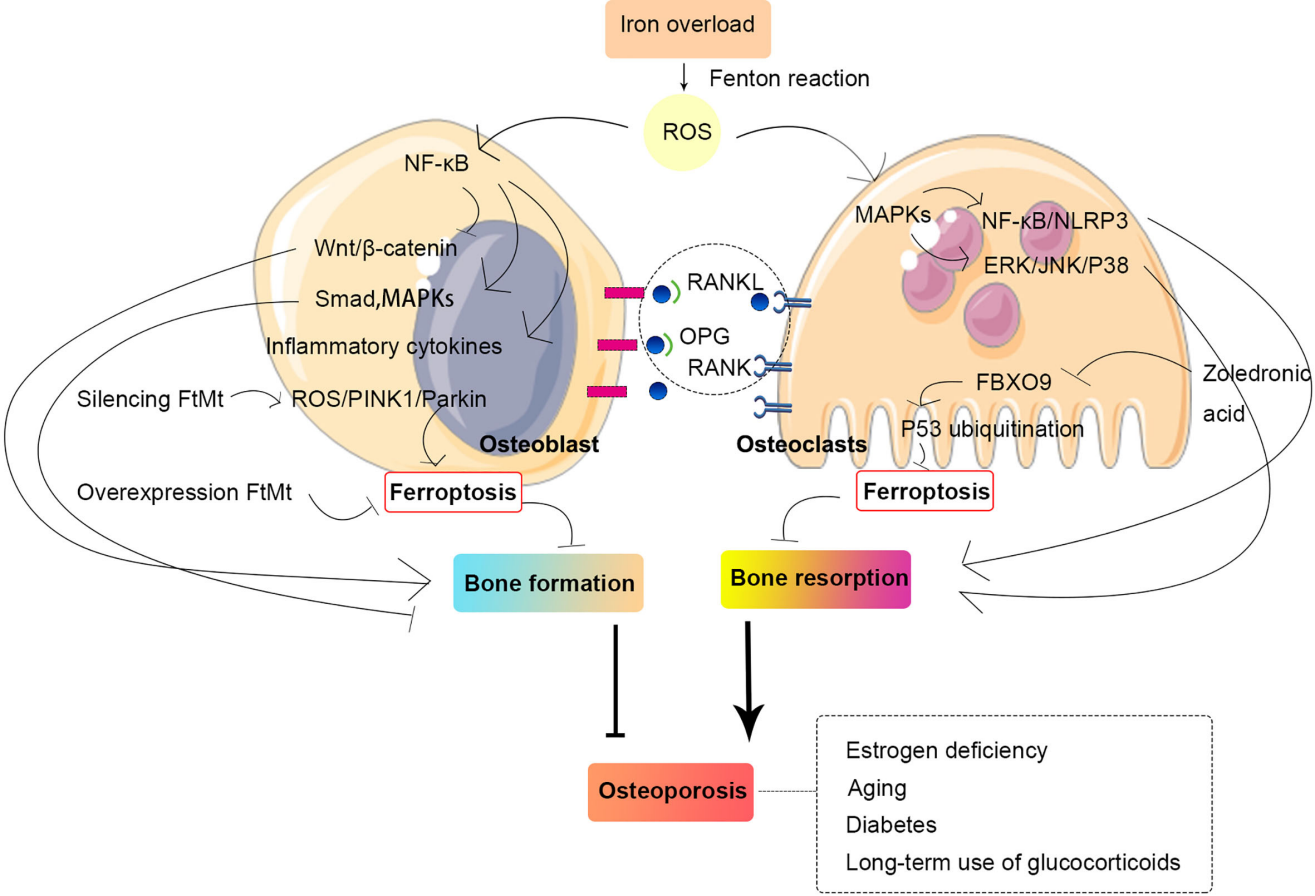
Relationship between osteoporosis and ferroptosis in osteoblast and osteoclast. The homeostasis and integrity of bone tissue require a balance between osteoclast and osteoblast activity. Iron overload can generate ROS through the Fenton reaction, and ROS can activate multiple intracellular signaling pathways, which, in turn, promote bone resorption and inhibit bone formation, thus leading to osteoporosis. Abbreviations: MAPK, mitogen-activated protein kinase; NF-κB, nuclear factor-kappa B; FtMt, mitochondrial ferritin; PINK1, PTEN-induced kinase 1; ROS, reactive oxygen species; OPG, osteoprotegerin; RANKL, receptor activator for nuclear factor-κB ligand; RANK, receptor activator for nuclear Factor-κB; NLRP3, NOD-like receptor family pyrin domain containing 3; ERK, extracellular signal–regulated kinase; JNK, c-Jun N-terminal kinase; FBXO9, F-box only protein 9.

Osteoporosis is a common disease and a major public health concern, which has heightened the fracture risk with an increasing prevalence in elderly people regardless of sex or age. The homeostasis and integrity of bone tissue require a balance between osteoclast and osteoblast activity. In addition, the remodeling of bone tissue is a continuous and cyclic process. In recent years, much attention has been focused on ferroptosis in the pathogenesis of osteoporosis (**Figure 4**).

### Ferroptosis occurs in osteoclasts

In terms of physiological characteristics, osteoclasts are multinucleated giant cells formed by the fusion of monocyte/macrophage precursor cells derived from myeloid progenitor cells in bone marrow with the indispensable involvement of macrophage colony-stimulating factor (M-CSF) and RANKL.

RANKL is stimulated by increasing the expression of the prostaglandin endoperoxide synthase 2 gene and MDA in bone marrow–derived macrophages (BMDMs) and decreasing GSH and iron levels, and iron accumulation is observed in mitochondria. ROS activate intracellular MAPK signaling pathways. ROS/MAPKs/nuclear transcription factor-kappa B (NF-κB)/NLRP3 activation causes osteoclast-mediated bone loss in diabetic osteoporosis (73). The activation of extracellular signal–regulated kinase, c-Jun N-terminal kinase (JNK), and P38 in the MAPK pathway can promote osteoclastogenesis, which leads to increased bone resorption (74). A recent study confirmed that zoledronic acid exerts ferroptosis-induced effects on osteoclasts by triggering FBXO9-mediated p53 ubiquitination and degradation (75). The inhibitory effect of artemisinin (ARS) compounds on osteoclast differentiation appears to be due to its downregulation of pathways involved in RANKL-induced osteoclastogenesis. In addition, mechanisms associated with intracellular iron, such as the cleavage of endoperoxide bridges, oxidative damage, and ferroptosis, are involved in the inhibition of osteoclast differentiation (76).

### Ferroptosis occurs in osteoblasts

Osteoblasts play an essential role in bone regeneration and play a leading role in the synthesis, secretion, and mineralization of the bone matrix (77). The inhibitory effect of iron on the osteogenic differentiation of MSCs has been described, and iron overload in mice is correlated with increased ferritin and decreased RUNX family transcription factor 2 (RUNX2) levels in compact bone osteoprogenitor cells (78).

NF-κB induces inflammatory factors, inhibits Wnt signaling, and activates Smad and MAPK signaling pathways in osteoblasts to inhibit osteogenic differentiation (79, 80). Mitochondrial ferritin (FtMt) is a protein that stores iron and intercepts toxic ferrous ions in cellular mitochondria. Many studies have shown that FtMt reduces oxidative stress and maintains intracellular iron homeostasis (81). The overexpression of FtMt reduces ferroptosis in osteoblasts under high-glucose conditions, whereas the silencing of FtMt can induce mitochondrial autophagy through the ROS/PINK1/Parkin pathway, which leads to increased ferroptosis in osteoblasts (82).

A previous study revealed that iron overload inhibits MC3T3 cell viability and induces apoptosis (83). This result suggests that iron overload may inhibit the activity of osteoblasts to some extent and thereby affects their differentiation and mineralization processes. Iron overload leads to excessive ROS and thus activates intracellular signaling pathways that affect cellular activity (84). A high dose of dexamethasone (10 μM) exerts its ferroptosis-induced effects on osteoblasts and thus downregulates GPX4 and system xc- (85). After induction with high glucose, MC3T3 cells, which exhibit increased ROS and reduced GPX4, have mitochondria that are generally smaller and less tubular, and the membrane exhibited darker staining and distinctly disrupted membrane folding. In a high-glucose environment, MC3T3 cells could the ability to reduce differentiation into osteoblasts and form mineralized nodules, as has similarly been observed in osteoblasts in mice (82, 86). In addition, it has been widely reported that advanced glycation end products play an important role in OP, particularly in diabetes−related OP, which may be caused by disruption of osteoblast functions by the induction of ferroptosis (87).

## Osteoarthritis

OA is a common disease that leads to pain, acute care hospitalizations, disability, and socioeconomic costs worldwide (88). Because of better understanding of its pathogenesis, the focus of treatment is shifting to the prevention and treatment at early stages. Studies have shown that OA does have some features in common with ferroptosis, such as lipid peroxidation (89), glutathione oxidation (90), mitochondrial dysfunction (91), and increased activities of lipoxygenase and cyclooxygenase in chondrocytes of OA cartilage (92).

Late-response upregulated genes are strongly enriched in the ferroptosis pathway in an *in vitro* model of OA treated with fibronectin fragments (FN-f) (93). A study pointed to a progressive increase in the sensitivity of chondrocytes to oxidative stress with decreases in GPX4, which suggests the role of GPX4 in ferroptosis in OA. In addition, GPX4 can aggravate extracellular matrix degradation through the MAPK/NF-κB pathway (94). Furthermore, Guo Z et al. (95) found that increased MMP13 expression and decreased collagen II expression in chondrocytes can be stimulated by chondrocyte ferroptosis.

**Figure 5:**
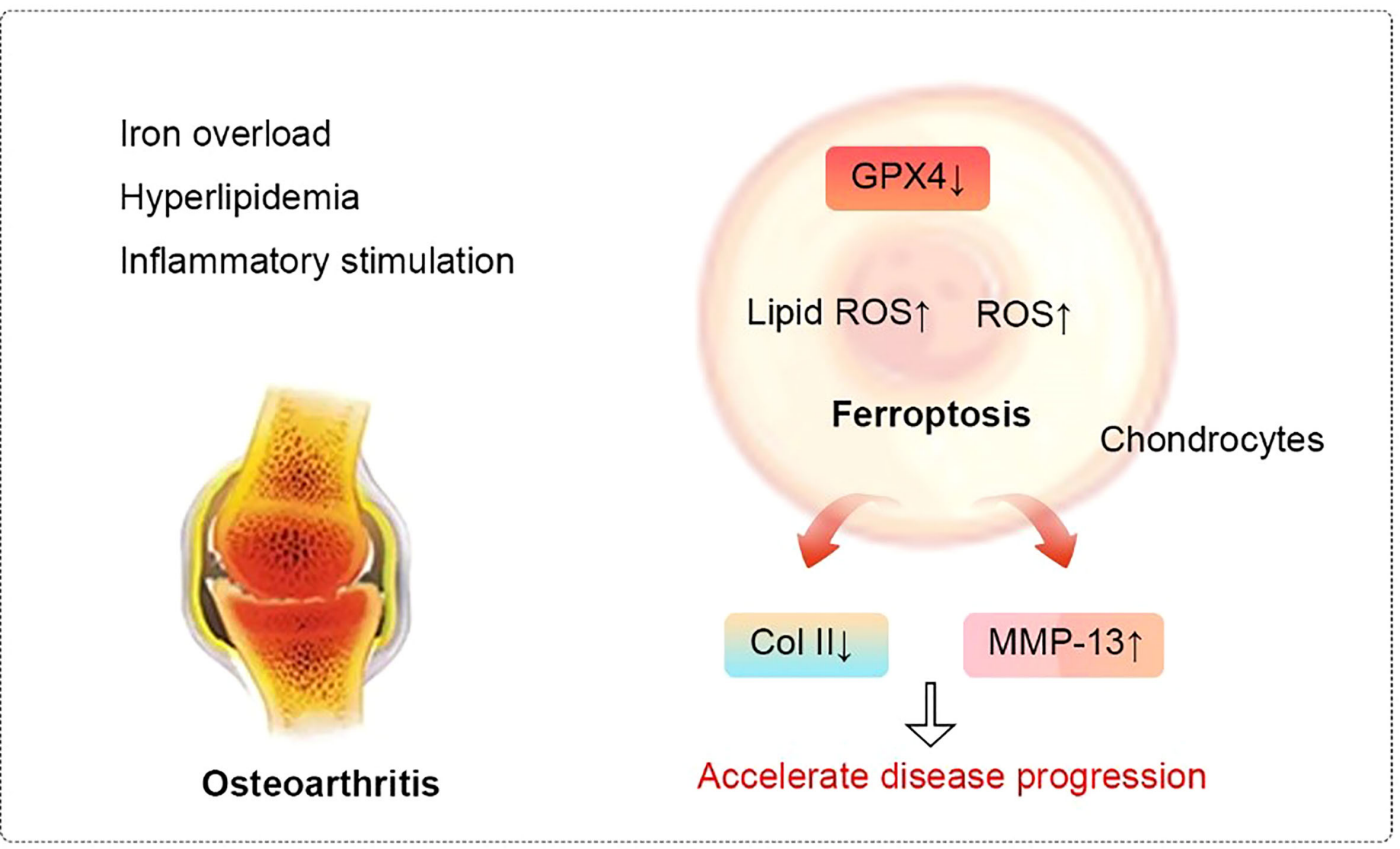
Relationship between ferroptosis and osteoarthritis. In cellular environments stimulated by iron overload, hyperlipidemia, and inflammation, the expression of Gpx4 in chondrocytes decreases. These changes lead to the accumulation of reactive oxygen species and lipid peroxides to ultimately induce ferroptosis. Ferroptosis, in turn, can progressively exacerbate the inflammatory response, leading to the increased expression of MMP-13 and decreased expression of collagen II in chondrocytes to accelerate the progression of OA. Abbreviations: ROS, reactive oxygen species; Col II, Type II collagen; MMP-13, matrix metalloproteinase-13.

Various novel treatments that inhibit ferroptosis are being extensively researched. For example, DFO has the ability to inhibit chondrocyte ferroptosis and promote NRF2 antioxidant system activation (96), which are essential for the protection of chondrocytes. In addition, activation of the system xc-/Gpx4 axis by icariin treatment can inhibit Gpx4, SLC7A11, and SLC3A2L expression and reduce TFR1 expression, which significantly reduces the induction of cell death and inhibits ferroptosis (97). Zhou et al. (98) found that D-mannose could alleviate OA progression *via* HIF-2α–mediated inhibition of the sensitivity of chondrocytes to ferroptosis. In addition, stigmasterol reduces IL-1β–induced damage and ferroptosis in ATDC5 cells through sterol regulatory element–binding transcription factor 2, which can also enhance the inhibitory effect of ferroptosis inhibitors on injury (99). The relationship between ferroptosis and OA is shown in **Figure 5**.

## Rheumatoid arthritis

RA is a relatively systemic common autoimmune disease with manifestations that include irreversible peripheral joint destruction and functional loss (100). Both genetic and environmental risk factors participate in the development of RA (101).

Oxidative stress and subsequent ROS-mediated cell death have recently been found to possibly play a critical role in RA development. Some evidence suggests that patients with RA with high persistent activity have reduced GPX activity in polymorphonuclear leucocytes (102). In addition, RSL3 can aggravate synovitis by inducing ferroptosis in synovial cells (97). Glycine can increase the S-adenosylmethionine (SAM) concentration to modulate the ferroptosis pathway by promoting SAM-mediated methylation of the GPX4 promoter and reducing FTH1 expression in RA fibroblast-like synoviocytes (103).

It has been indicated that serotransferrin-related molecules may be a promising method for investigating refractory RA (104). In addition, Tumor necrosis factor (TNF) signaling exerts its effect on fibroblasts in different manners to protect them from ferroptosis, such as promoting cystine uptake and increasing the biosynthesis of GSH (105). Moreover, studies have shown that CoQ10, a fat-soluble antioxidant, is a crucial regulator of ferroptosis. Jhun et al. (106) used CoQ10-encoded liposome/gold hybrid nanoparticles to attenuate RA progression by targeting signal transducer and activator of transcription-3/T helper cell 17 (STAT3/Th17).

## Intervertebral disc degeneration

Intervertebral disc degeneration (IVDD) is a complex pathological condition caused by intractable back pain or secondary neurological deficits involving age-related changes and tissue damage produced by multiple stresses (107, 108). Because there is currently no fundamental treatment for the disease, an indirect symptom relief strategy has been employed. Of note, increasing evidence suggests that ferroptosis is involved in IVDD, which provides a new direction for therapeutic targets.

Zhang et al. (109) previously demonstrated that ferroptosis is involved in IVDD. Ferroptosis in related tissue may appear to be due to exposure to high levels of heme, which may be caused by neovascularization in the prominent nucleus pulposus, and the progressive degeneration of herniated nucleus pulposus is also accelerated (110). Moreover, the levels of GPX4 and FTH1 in the degenerated disc tissues of IVDD rats are lower than those in those of healthy rats (111). Studies have also shown that the pathogenesis of IVDD involves ferritin degradation mediated by ferritin phagocytosis and subsequent lipid peroxidation. The disruption of iron homeostasis in degenerative disc tissue may be driven by increased levels of autophagy and NCOA4‐regulated ferritinophagy upon exposure to tert-butyl hydroperoxide. In addition, homocysteine, as a novel contributor to IVDD, exerts its effects on oxidative stress and ferroptosis in the nucleus pulposus by enhancing GPX4 methylation (112).

An increase in the nuclear translocation of metal-regulated transcription factor 1 is achieved by restoring FPN function, eliminating intercellular iron overload, and thus protecting cells from ferroptosis. In addition, the process can be enhanced by hinokitiol through inhibition of the JNK pathway, which results in improving the progression of IVDD *in vivo* (113). It has been confirmed that IL-6 and its receptor led to chondrocyte ferroptosis by inducing cellular oxidative stress and interfering with iron homeostasis, which can be inhibited by overexpression of miR-10a-5p, and these findings suggest that the IL-6/miR-10a-5p/IL-6R axis could be a potential therapeutic target for intervention in IVDD (114).

## Sarcopenia

Sarcopenia is defined as low muscle mass together with low muscle function. Diseases that can lead to secondary sarcopenia include malignancies, chronic obstructive pulmonary disease, heart failure, and kidney failure. Thus, further research is needed for the development of appropriate methods for the management of sarcopenia (115).

Soaring evidence supports the role of ROS accumulation and decreased endogenous antioxidant mechanisms in the progression of sarcopenia (116–118). Previous studies have reported associations among muscle iron accumulation, ROS production, and muscle wasting (119–121). Studies have also revealed that skeletal muscle atrophy induced by iron overload is related to ROS-mediated ubiquitin–proteasome system activation (122). Notably, iron plays a crucial role in ferroptosis triggered by P53-Slc7a11 in muscles, which suggests a therapeutic strategy for targeting iron accumulation (123). Interestingly, the release of iron mediated by macrophages can facilitate muscle regeneration (124), whereas oxidative stress and skeletal muscle atrophy have been found in mice with chronic iron injection for 14 days (125). In addition, supplementation with DFO, an iron chelating agent, has the ability to reduce oxidative stress and inflammation in the diaphragm muscle of mice with Duchenne dystrophy (126). The latest research has shown that satellite cell–specific deletion of TFR1 impairs skeletal muscle regeneration by activating ferroptosis (127). Given the effect of iron on ROS production, the role of iron in the homeostasis of muscle satellite cells deserves more attention.

## Rhabdomyolysis

Rhabdomyolysis (RM) is a common disease associated with myoglobinuria, electrolyte abnormalities, and acute kidney injury (AKI). The aims of the related treatments are to stop further skeletal muscle damage, prevent acute renal failure, and rapidly detect potentially life-threatening complications (128, 129).

**Figure 6:**
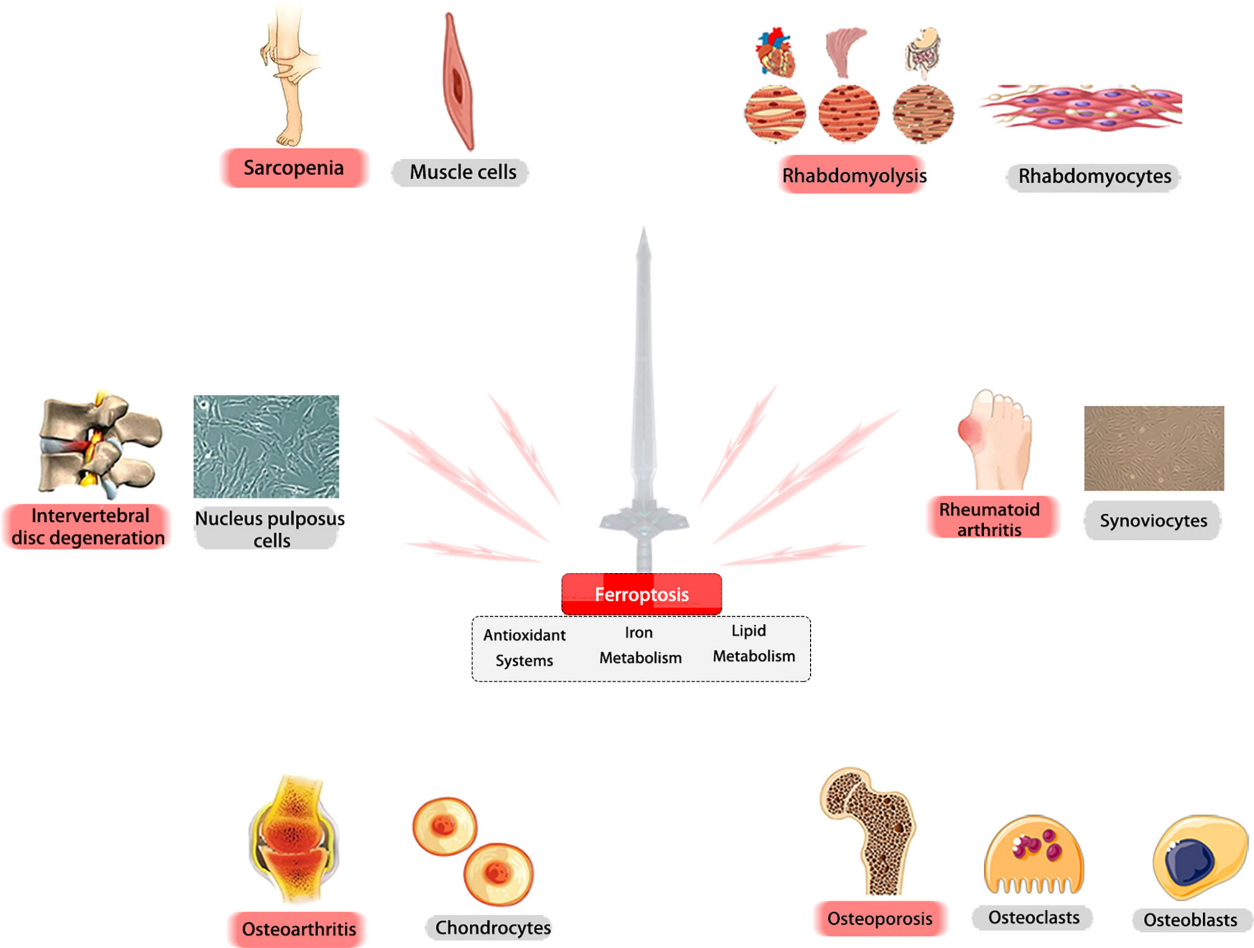
Ferroptosis may play a vital role as a fundamental mechanism in a variety of MSKs. Ferroptosis is critically involved in the pathogenesis of various MSKs. In different tissues and cells, ferroptosis -related molecules could be promising targets for treating these diseases.

A recent study has implicated ferroptosis in RM-related kidney damage *in vivo* and *in vitro* (130). RM results in the release of muscle cell components, including myoglobin, into the bloodstream, and the resulting myoglobin is freely filtered by the glomerulus and reabsorbed by the proximal tubule, which results in the promotion of ferroptosis-mediated cell death and leads to AKI. Moreover, the severity of AKI can be inhibited by Fer-1 treatment through the reduction of myoglobin-derived iron accumulation and lipid peroxidation (131). In animal models, iron chelators also have the ability to protect functional and histologic RM (132). Another previous study indicated the protective role of ACSL4 in mediating ferroptosis in the development of RM following EHS, which suggests that ACSL4 may also be a novel therapeutic target in RM (133). Overall, we point out that ferroptosis may play a vital role as a fundamental mechanism in a variety of MSKs (**Figure 6**).

## Discussion

With advancing aging, the burden of MSKs will undoubtedly increase. In addition, the prevalence of people not paying attention to their diet, lifestyle, health, and physical activity contributes to the disease burden (134). Unclear pathogenic mechanisms for age-related MSKs challenge clinical practitioners (135). Raising the public awareness of risk factors and increasing their understanding by the medical and scientific community are pragmatic approaches to address these issues.

Ferroptosis, a novel and unique form of RCD, is broadly involved in the development of cancers, kidney diseases, neurological diseases, and MSKs. In this review, we summarized the classic pathways of ferroptosis, including iron metabolism, antioxidant systems, and lipid metabolism, with a focus on affected disorders such as osteoporosis, OA, RA, IVDD, sarcopenia, and RM. Of note, iron, lipids, and ROS play an irreplaceable role in cell survival. However, excessive dependence is a double-edged sword. These three factors maintain normal body function in a steady state and strike a deadly blow to cells when metabolic disorders occur. The complex biological processes involved in ferroptosis are induced by an imbalance among antioxidants, iron, and lipid dynamics, but their role and contribution to the occurrence and metastasis of MSKs remain unclear.

Exploring the underlying mechanisms of ferroptosis in MSKs appears to have diverse favorable effects (136); however, before we move to related clinical applications, various issues need to be addressed. First, the fact that the current studies on ferroptosis and MSKs only scratch the surface of phenomena and results remains a challenge for precision medicine, and the detailed role of ferroptosis in the occurrence and development of MSKs has not been thoroughly studied. Second, most studies of MSKs have investigated ferroptosis only in cells and animal models and lack validated clinical evidence. Thus, clinical studies are imperative to solidify a conclusive role of ferroptosis in humans as soon as possible. Finally, ferroptosis appears to be a double-edged sword for MSKs. The inhibition of ferroptosis relieves the symptoms of osteoporosis or OA. However, the toxicity of ferroptosis-inhibiting or ferroptosis-inducing compounds on other organs is largely unclear. This evidence indicates that the role of ferroptosis is different in MSKs. Hence, although several characteristics and biomarkers of ferroptosis have been proposed, the specific and accurate quantification of the ferroptosis response, particularly *in vivo*, remains a major challenge.

In summary, ferroptosis is critically involved in the pathogenesis of various MSKs. With ongoing research, we point out that ferroptosis will be an area worthy of in-depth study and will play a vital role in the subsequent development and clinical translation of new drugs for MSKs.

## Author contributions

XW and LZ were responsible for the conceptualization of the study. XH was responsible for the methodology. XH and YZ were responsible for the validation. XH and YZ completed the formal analysis. CS, BQ, and NL were responsible for the resources. KS completed the data curation. YZ and XH prepared the original draft. XW and LZ reviewed and edited the manuscript. All authors contributed to the article and approved the submitted version.

## Funding

This study was supported by the National Natural Science Foundation of China (No. 82205140), Basic Research Program of Jiangsu Province (Natural Science Foundation) (No. BK20220468), the Innovation Team and Talents Cultivation Program of the National Administration of Traditional Chinese Medicine (No. ZYYCXTD-C-202003) and the Fundamental Research Funds for the Central Public Welfare Research Institutes (No. ZZ13-YQ-039).

## Conflict of interest

The authors declare that the research was conducted in the absence of any commercial or financial relationships that could be construed as a potential conflict of interest.

## Publisher’s note

All claims expressed in this article are solely those of the authors and do not necessarily represent those of their affiliated organizations, or those of the publisher, the editors and the reviewers. Any product that may be evaluated in this article, or claim that may be made by its manufacturer, is not guaranteed or endorsed by the publisher.

## Glossary

**Table:**

MSKs	Musculoskeletal disorders
ACD	Accidental cell death
RCD	Regulated cell death
ROS	reactive oxygen species
OA	Osteoarthritis
RA	Rheumatoid arthritis
HO·	Hydroxyl radicals
TFR1	Transferrin receptor 1
STEAP3	Six-transmembrane epithelial antigen of prostate 3
DMT1	Divalent metal transporter 1
LIP	Labile iron pool
NCOA4	Nuclear receptor coactivator 4
FPN1	Ferroportin 1
FTH1	Ferritin heavy chain 1
FTL1	Ferritin light chain 1
Fer-1	Ferrostatin-1
DFO	Defetoxamine
GPX4	Glutathione peroxidase 4
GSH	Glutathione
GSSG	Oxidized glutathione
L-OOH	lipid peroxides
LOH	lipid hydroxyl derivatives
RSL3	RAS-selective-lethal-3
ATF4	Activation of transcription factor 4
system xc-	the cystine/glutamate antiporter system
NRF2	NF-E2-related factor 2
BAP1	BRCA-1-associated protein
ATF3	The activation of transcription factor 3
BECN1	beclin 1
TCA	Tricarboxylic acid
PUFAs	Polyunsaturated fatty acids
4-HNE	4-hydroxynonenal
MDA	Malondialdehyde
MUFAs	Monounsaturated fatty acids
ACSL3	acyl-coenzyme A synthetase long-chain family member 3
Lip-1	liproxstatin-1
FSP1	Ferroptosis-suppressor-protein 1
NADPH	Nicotinamide adenine dinucleotide phosphate
BH4	tetrahydrobiopterin
GCH1	GTP cyclohydrolase-1
M-CSF	Macrophage colony-stimulating factor
PTGS2	Prostaglandin endoperoxide synthase 2
BMDMs	Bone marrow-derived macrophages
DOP	Diabetic osteoporosis
ARS	Artemisinin
RUNX2	RUNX family transcription factor 2
FtMt	Mitochondrial ferritin
AGEs	Advanced glycation end products
FN-f	Fibronectin fragments
ECM	Extracellular matrix
STM	Stigmasterol
SREBF2	Sterol regulatory element binding transcription factor 2
SAM	S-adenosylmethionine
FLSs	Fibroblast-like synoviocytes
STAT3/Th17	Signal transducer and activator of transcription-3/T helper cell 17
IVDD	Intervertebral disc degeneration
MTF1	Metal-regulated transcription factor 1
AKI	Acute kidney injury
